# Complete plastome sequence of *Hoya carnosa* (L. f.) R. Br. (Apocynaceae)

**DOI:** 10.1080/23802359.2019.1710596

**Published:** 2020-01-17

**Authors:** Xue-Fen Wei, Si-Jin Zeng, Guo-Qiang Zhang, Guang-Da Tang, Jiu-Xiang Huang

**Affiliations:** a College of Forestry and Landscape Architecture, South China Limestone Plants Research Center, South China Agricultural University, Guangzhou, China;; b Shenzhen Key Laboratory for Orchid Conservation and Utilization, The National Orchid Conservation Centre of China and The Orchid Conservation and Research Centre of Shenzhen, Shenzhen, China

**Keywords:** *Hoya carnosa*, plastome genome, Apocynaceae

## Abstract

*Hoya* is a remarkable genus with high horticultural ornamental value. In this study, we report and characterize the complete plastid genome sequence of *Hoya carnosa.* The complete chloroplast genome was 176,340 bp in length, which includes a pair of inverted repeat regions (IRs) of 41,381 bp separated by a large single copy region (LSC) 91,281 bp and a small single copy region (SSC) 2,297 bp. Interestingly, IRs expanded into SSC, with the result that most of the genes in SSC were duplicated. This chloroplast genome contained 110 genes, including 76 protein-coding genes, 30 tRNA genes, and 4 rRNA genes. The complete plastome sequence of *H. carnosa* will provide some useful information for future phylogenetic study of *Hoya* and its horticultural application.

As one of the most complex and sophisticated groups of flowering plants, *Hoya* (Asclepiadoideae, Apocynaceae) is a remarkable genus with high horticultural ornamental value (Wanntorp et al. 2006). At least 200 species are currently recognized within Apocynaceae and the list of the species of *Hoya* is updated (Wanntorp et al. 2006; Rahayu and Rodda 2019). Most species of *Hoya* are vines, often twining or climbing with adventitious roots (Li et al. 1995). However, the complete classification system is lacking for the genus although some molecular phylogeny has been done (Tan et al. 2018). The information on the chloroplast data obtained is still far from enough to solve the relationship between species within the genus.

In this study, *Hoya carnosa* was sampled from National Orchid Conservation Center in Guangdong province of China (114°19′01″E, 22°60′34″N) and the specimen (voucher SZ708) was deposited at the South China Agricultural University Herbarium (CANT). DNA was acquired from the young leaves of its plant and the total genome was sequenced using Illumina HiSeq 2000 platform. Total clean reads were aligned to the chloroplast genome from the related species *H. liangii* (GenBank accession: MH678666) and *H. pottsii* (MH678667), then assembled using the GetOrganelle Tookit (Jin et al. 2018). Annotation of the chloroplast genomes was carried out using Plastid Genome Annotator (Qu et al. 2019) and Geneious Prime 2019 (https://www.geneious.com). This newly obtained plastome was submitted to GenBank (ID: MN781974).

The complete chloroplast genome of *H. carnosa* was a circular molecule of 176,340 bp long, which includes two inverted repeat regions (IRs) of 41,381 bp, separated by a large single copy region (LSC) 91,281 bp and a small single copy region (SSC) 2,297 bp. Interestingly, IRs expanded into SSC, with the result that most of the genes in SSC were duplicated. The chloroplast genome contained 110 genes, including 76 protein-coding genes, 30 tRNA genes, and 4 rRNA genes. The GC content of the whole plastome was 37.1%.

We used RAxML (Stamatakis 2014) with 1000 bootstraps under the GTR + G substitution model to reconstruct a maximum likelihood (ML) phylogeny of *H. carnosa* with 18 published complete plastome of Apocynaceae. Two species, *Gentiana straminea* (Gentianaceae) and *Coffea arabica* (Rubiaceae), were used as outgroups. The phylogenetic tree showed that *H. carnosa* was the sister of *H. liangii*, and three *Hoya* species formed a single clade (Figure 1). The complete plastome sequence of *H. carnosa* will provide useful information for phylogenetic studies and horticultural application in *Hoya.*

**Figure 1.**
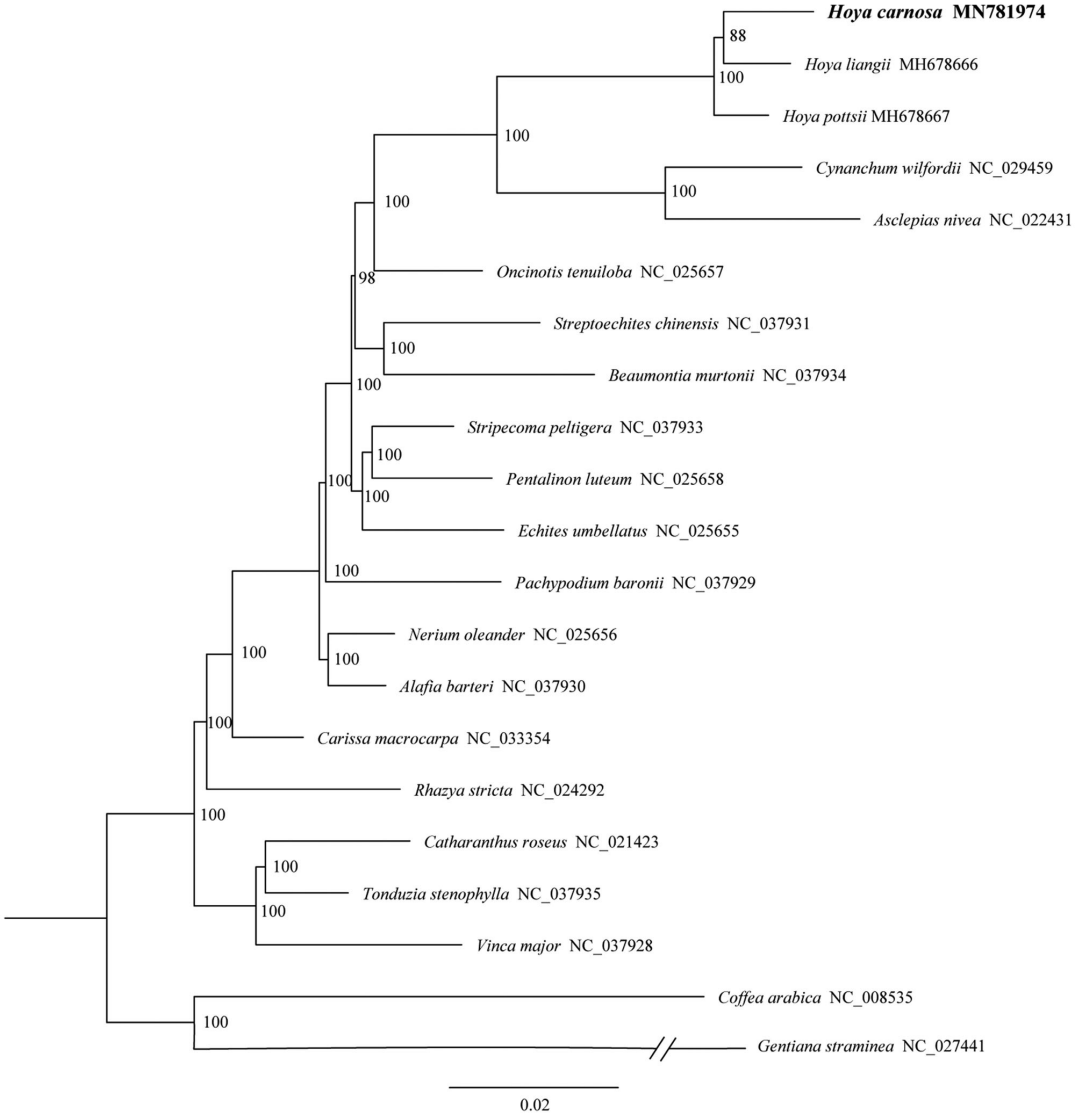
Maximum-likelihood tree from the analysis of nucleotide substitutions of 18 complete chloroplast genome of Apocynaceae, with Gentiana straminea (Gentianaceae) and Coffea arabica (Rubiaceae) as outgroup. Numbers on branches are bootstrap support values.
